# Sanguinarine inhibits growth and invasion of gastric cancer cells *via* regulation of the DUSP4/ERK pathway

**DOI:** 10.1111/jcmm.13043

**Published:** 2016-12-13

**Authors:** Rui Zhang, Ge Wang, Peng‐Fei Zhang, Jing Zhang, Yan‐Xia Huang, Yun‐Min Lu, Wei Da, Qun Sun, Jin‐Shui Zhu

**Affiliations:** ^1^ Department of GastroenterologyShanghai Jiao Tong University Affiliated Shanghai Sixth People's HospitalShanghaiChina

**Keywords:** Sanguinarine, dual‐specificity phosphatase 4, extracellular signal‐regulated kinase, gastric cancer, proliferation, invasion

## Abstract

Sanguinarine, a bioactive benzophenanthridine alkaloid extracted from plants of the Papaveraceae family, has shown antitumour effects in multiple cancer cells. But the therapeutic effects and regulatory mechanisms of sanguinatine in gastric cancer (GC) remain elusive. This study was aimed to investigate the correlation of dual‐specificity phosphatase 4 (DUSP4) expression with clinicopathologic features and overall survival in patients with GC and explore the effects of sanguinarine on tumour growth and invasion in GC cells (SGC‐7901 and HGC‐27) and underlying molecular mechanisms. Immunohistochemical analysis showed that decreased DUSP4 expression was associated with the sex, tumour size, depth of invasion and distant metastasis in patients with GC. Functional experiments including CCK‐8, Transwell and flow cytometry analysis indicated that sanguinarine or DUSP4 overexpression inhibited GC cell viability and invasive potential, and induced cell apoptosis and cycle arrest in S phase, but DUSP4 knockdown attenuated the antitumour activity of sanguinarine. Further observation demonstrated that sanguinarine up‐regulated the expression of DUSP4 and Bcl‐2‐associated X protein (Bax), but down‐regulated phosphorylated extracellular signal‐regulated kinase (p‐ERK), proliferating cell nuclear antigen (PCNA), matrix metalloproteinase 2 (MMP‐2) and B‐cell lymphoma 2 (Bcl‐2) expression. Taken together, our findings indicate that sanguinarine inhibits growth and invasion of GC cells through regulation of the DUSP4/ERK pathway, suggesting that sanguinarine may have potential for use in GC treatment.

## Introduction

GC, an aggressive form of digestive system tumours, has the third highest lethality and fourth highest morbidity in all cancers worldwide 1. Japanese Gastric Cancer Association (JGCA) has identified two clinical variants: early gastric cancer (EGC) and advanced GC. EGC refers to GC limited to the mucosa or submucosa, regardless of the tumour size and lymph node metastasis. Relatively, when carcinoma tissue infiltrates into the muscular layer, or serosa layer of stomach, it turns into the advanced GC 2. More than 80% of the diagnosis is made at the advanced stages of GC 3.

The prognosis of advanced GC is poor, as its overall survival (OS) is less than 12 months 4. Currently, first‐line palliative chemotherapy, especially combination regimens (two‐drug or three‐drug cytotoxic regimens), plays an important role in the treatment of advanced GC 5. However, the objective response rate of first‐line chemotherapy in unresectable advanced GC (local or metastatic) is unsatisfactory, lingering at 25–55%. The median progression‐free survival (PFS) is 4–7 months, and the OS is 9–14 months. No significant improvement is made even with the addition of the targeted drug trastuzumab 6, 7. Therefore, development of more effective chemotherapeutic agents to improve therapy for GC is urgently needed.

The dual‐specificity phosphatases (DUSPs) are a family of 25 phosphatases, which can dephosphorylate both tyrosine and serine/threonine residues. DUSP4 is a member of DUSPs family and is also named as mitogen‐activated protein kinase phosphatase 2 (MKP2). Mitogen‐activated protein kinase (MAPK), which can be inhibited by dephosphorylation of a critical motif in the kinase domain, is the primary substrate of the DUSPs 8. The MAPK signalling pathway is evolutionarily conserved 9 and can be activated by multiple extracellular stimuli, such as growth factors, cytokines and environmental factors, and then induces a series of intracellular responses, including the regulation of gene expression, cell proliferation, apoptosis and metabolism 10, 11. Extracellular signal‐regulated kinase (ERK) is one of the earliest identified mammalian MAPK genes. ERK regulates cellular mobility in GC cell lines through mediating the activity of matrix metalloproteinases (MMPs) 12, 13, 14. DUSP4 inactivates MAPKs (ERK, p38 and JNK) by dephosphorylating them in cell lines 15, thus exerting an important role in cell physiology and pathological processes, such as cell growth, cellular senescence, stress‐induced apoptosis and cancers 16. Genomic DUSP4 loss is a frequent event in many cancer types, indicating that DUSP4 may have tumour‐suppressive function 17.

Traditional herbal medicines, containing various natural biologically active compounds, have therapeutic efficacy with minimal adverse effects, providing sources for developing first‐line drugs 18, 19. Sanguinarine is a bioactive benzophenanthridine alkaloid found in plants of the Papaveraceae family 20. Previous studies have found that sanguinarine has broad‐spectrum pharmacological properties, including anti‐inflammatory, antimicrobial, antioxidative and anaesthesia effects on the central nervous system 21. Recently, its anticancer properties have been testified in various cancers 20, 22, 23, 24, 25. Some studies show that DUSP4 is significantly up‐regulated by sanguinarine in pancreatic cancer cells, suggesting that sanguinarine may act as the DUSP4 activator exerting its activity in cancer 20. However, there is little study about sanguinarine effects in GC. Here, our studies show that sanguinarine inhibits growth and invasion, and induces apoptosis and cycle arrest in human GC cells through regulation of the DUSP4/ERK pathway, indicating the promising preclinical activity for treatment of GC.

## Materials and methods

### Reagents and compounds

Sanguinarine (purity ≥98%) was purchased from Dalian Meilun Biotechnology Co., Ltd. (Dalian, China). Cell Counting Kit‐8 (CCK‐8) was purchased from Shanghai Beyotime Biotechnology Co., Ltd. (Shanghai, China). All the supplies of cell culture were purchased from Thermo Fisher Scientific Company (Waltham, MA, USA). Human GC tissues and the corresponding adjacent non‐cancerous tissues (ANCT) were collected from Shanghai Jiao Tong University affiliated Shanghai Sixth People's Hospital. GC tissue microarray was made by Shanghai Outdo Biotech CO. LTD (Shanghai, China). GC cell lines (GES‐1, SGC‐7901, BGC‐823, HGC‐27, AGS and MGC‐803) used in these experiments were from Laboratory of Gastroenterology of Shanghai Sixth People's Hospital. Lentivirus‐mediated DUSP4 overexpression vector, lentivirus‐mediated DUSP4 siRNA vector (si‐DUSP4), negative control vector (NC) and virion‐packaging elements were purchased from Genechem (Shanghai, China); All antibodies used in this study were purchased from Cell Signaling Technologies (Beverly, MA, USA).

### Tissue microarray analysis

Human GC tissue microarray (HStm‐Ade180Sur‐04, 89 cases, Outdo Biotech) was used to detect the levels of DUSP4 expression in GC and ANCT tissues. DUSP4 expression was semi‐quantitatively evaluated by immunohistochemical staining. Stained intensity and percentage of stained cells were considered as the standards of DUSP4 expression degree. Stained intensity was graded as non‐staining (0), light yellow (1), brownish yellow (2) and sepia (3), and percentage of stained cells was graded as <5% (0), 5–25% (1), 26–50% (2), 51–75% (3) and >75% (4). The final score (0–12) was product of stained intensity and percentage of stained cells. Samples had score<4 were classified as low expression, and samples had score 4–12 were classified as were high expression.

### Clinical samples and data

Tumour tissues were collected from biopsy samples undergoing resection of the primary GC in a total of 89 consecutive cases admitted in our hospital from May 2007 to August 2013. The baseline characteristics of the patients before neo‐adjuvant chemotherapy were summarized. Follow‐up studies included physical examination, laboratory analysis and computed tomography if necessary. Overall survival was defined as the interval between the dates of surgery and death. The study was approved by Medical Ethics Committee of Shanghai Jiao Tong University, and written informed consent was obtained from the patients or their parents before sample collection.

### Cell culture and transfection

Human GC cells (SGC‐7901, BGC‐823, HGC‐27, AGS and MGC‐803) and GES‐1 were cultured in DMEM supplemented with 10% foetal bovine serum (FBS), penicillin (100 U/ml) and streptomycin (100 μg/ml) at 37°C in an incubator with humidified atmosphere of 95% air and 5% CO_2_. When the cells were grown to 60–70%, old medium was removed, and FBS‐free medium containing lentivirus‐mediated siRNAs targeting DUSP4 (sequence #9: Thermoscientific cat#, J‐003963‐09) was added into the culture dishes. After culturing for 24 hrs, medium containing lentivirus was replaced by DMEM supplemented with 10% FBS.

### Cell proliferation‐toxicity assay

GC cells (2 × 10^3^/100 μl/well) were seeded in 96‐well plates and incubated for 15 hrs (37°C, 5% CO_2_). Then, sanguinarine diluted by complete medium (100 μl/well) at serial concentrations (0/5/10/30 μmol/l) was added to each well. After treating for 24, 48, 72 or 96 hrs, CCK‐8 solution (10 μl) was added into each well, followed by incubation for 1 hr. The optical densities at 450 nm were measured using a Microplate Reader (Molecular Devices Sunnyvale, CA, USA).

### Cell cycle analysis

GC cells were collected after incubating with different concentrations of sanguinarine (0/5/10/30 μmol/l) for 48 hrs, and then, the cells were placed at 4°C for 12 hrs after adding 70% ethanol to them. Cells were centrifuged (1509 g, 5 min.), and then washed with PBS, followed by staining with propidium iodide (PI, 1 ml). After placing the cells in the dark at 4°C for 20 min., the cell cycle distribution was analysed by a FC 500 flow cytometer (Beckman, 250 S. Kraemer Boulevard Brea, CA, USA).

### Cell apoptosis analysis

Apoptosis analyses were conducted by the Annexin V–FITC Apoptosis Detection Kits (Beyotime, Shanghai, China). GC cells were collected after incubating with different concentrations of sanguinarine (0/5/10/30 μmol/l) for 48 hrs, followed by being washed with PBS for three times. Then, resuspending cells were mixed with binding buffer (100 μl). Annexin V‐FITC (5 μl) and PI (5 μl) were added to the cells. After incubating for 10 min. at the room temperature in the dark, the cell apoptosis analysis was analysed by flow cytometry.

### Cell invasion assay

Cell invasion assays were performed by the Transwell chambers (Beyotime) with polycarbonate membrane (8 μm). Polycarbonate membrane was pre‐coated with Matrigel (5 mg/ml) and rehydrated with FBS‐free DMEM medium (50 μl, 30 min., 37°C). Then, cells (5 × 10^5^/ml) collected from different concentrations of sanguinarine (0/5/10/30 μmol/l) were placed in the upper chambers with FBS‐free DMEM medium (400 μl), while DMEM medium with 10% FBS (600 μl) was placed in the lower chambers. Non‐invaded cells on the upper surface were wiped by cotton swabs after incubation for 24 hrs, while invaded cells on the lower surface were fixed (30 min., 4% paraformaldehyde) and stained (haematoxylin–eosin, HE). Five fields were selected for counting in each sample. Results were expressed as the average number of migrated cells.

### Reverse transcriptase polymerase chain reaction (RT‐PCR)

Total RNA from cells of experimental groups was extracted according to the instructions, and then, total RNA was reversely transcribed to cDNA by a RT‐PCR Kit (Takara, Dalian, China). The amplification primers were designed and synthesized by Shanghai Genechem Co., Ltd. (Table S1). PCR was performed by an ABI 7500 PCR instrument. The amplification reaction condition was as follows: 95°C for 30 sec., 60°C for 30 sec. and 72°C for 60 sec. This procedure was repeated for 30 cycles. PCR products were conducted electrophoresis on the Sepharose gel, photographed by gel document system and analysed by Quantity One software (Bio‐Rad, Hercules, CA, USA).

### Western blot analysis

Cells (5 × 10^6^) in logarithmic growth phase were collected and lysed for total proteins. The supernatant fluid of lysate was collected by centrifugation, followed by SDS‐PAGE electrophoresis. After electrophoresis, the proteins were electro‐transferred onto a polyvinylidene fluoride (PVDF; Millipore Boston, MA, USA) membrane. The membrane was then rinsed with a blocking solution of 5% non‐fat milk for 60 min. and incubated overnight at 4°C with antibodies against DUSP4, p‐ERK, PCNA, MMP‐2 and Bcl‐2, followed by incubation with horseradish peroxidase‐conjugated secondary antibodies at room temperature for 1 hr. All protein expression levels were normalized to the level of β‐actin expression.

### Statistical analysis

Experimental data were dealt with software SPSS 19.0 (IBM, Armonk, NY, USA). The values from at least three independent experiments were recorded as mean ± S.E.M. Student's *t‐*test was used for comparisons between two measurement data, analysis of variance was used for comparisons among three groups, and chi‐square test was used for comparisons of count data. Kaplan–Meier method was used to determine OS. The differences were statistically significant when *P* values were less than 0.05.

## Results

### The expression of DUSP4 in GC tissues and cell lines

**Figure 1:**
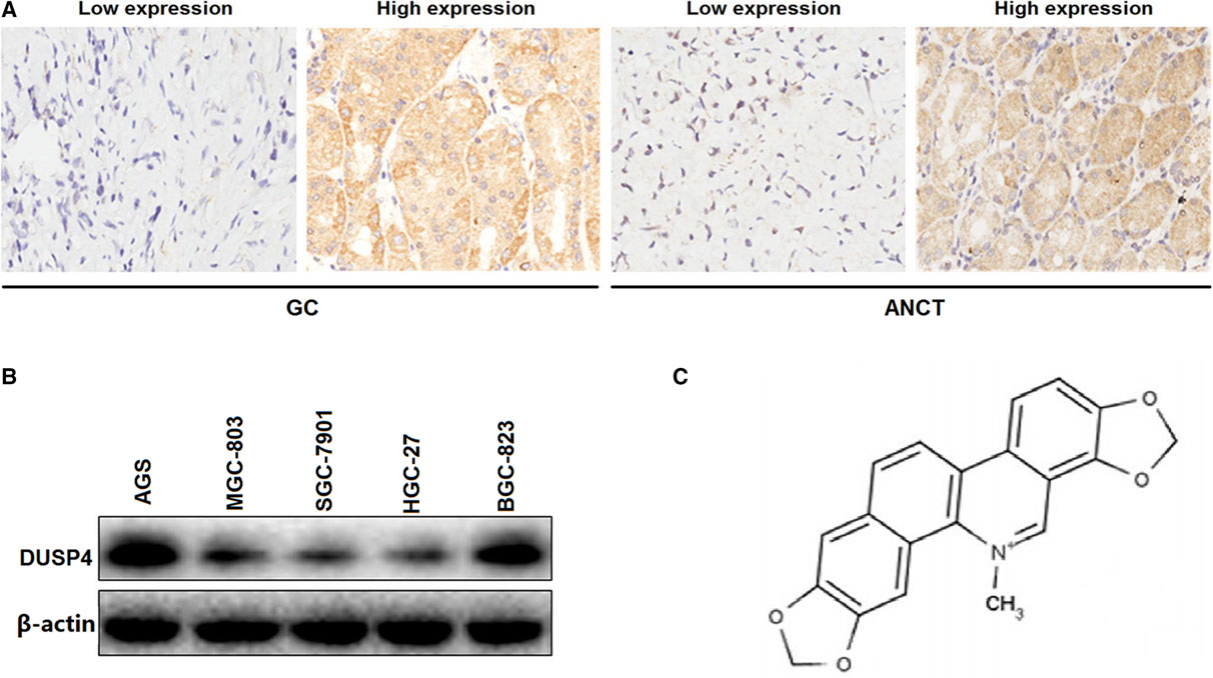
The expression level of DUSP4 in GC tissues and cell lines. (**A**) Representative microphotographs of DUSP4 immunohistochemical staining in GC and ANCT tissues (×200). (**B**) The protein expression levels of DUSP4 in GC cell lines. (**C**) The chemical structure of sanguinarine.

To examine the expression of DUSP4 in GC tissues, we detected its expression level in 89 cases of GC patients with paired ANCT by IHC. In those cases, various grades of cytoplasmic DUSP4 expression were observed, and four representative photomicrographs were shown in Figure 1A. DUSP4 expression level was found low in 44 cases (49.4%) of GC tissues and 24 cases (27.0%) of ANCT tissues (*P* = 0.001, Table S3). The survival curves demonstrated that DUSP4 expression had no significant correlation with the OS in patients with GC (*P* = 0.205, Supplemental figure). In addition, the protein expression of DUSP4 was detected in different GC cell lines (AGS, MGC‐803, SGC‐7901, HGC‐27 and BGC‐823) by Western blotting, indicating that DUSP4 expression level was markedly down‐regulated in SGC‐7901 and HGC‐27 cell lines compared with other ones (Fig. 1B).

### Association of DUSP4 expression with clinicopathologic features and prognosis in GC patients

The correlation between DUSP4 expression and some clinicopathological parameters was investigated to assess the clinical significance of DUSP4 expression in GC (Table 1). The results showed that decreased DUSP4 expression was correlated with gender (*P* = 0.037), tumour size (*P* = 0.020), depth of invasion (*P* = 0.008) and distant metastasis (*P* = 0.016). However, DUSP4 expression had no correlation with age, AJCC (American Joint Committee on Cancer) stage, T stage and N stage (*P* > 0.05, Table 1). Kaplan–Meier and COX regression analysis were used to assess the association of DUSP4 expression with OS in patients with GC (Table S2). KM method showed that tumour size (*P* < 0.001) and AJCC stage (*P* < 0.001) affected the OS, but DUSP4 expression had no correlation with OS. However, if the survival time was divided into ≤40 and >40 months, we found that DUSP4 high expression was correlated with better short‐term prognosis (within 3 years, *P* = 0.049) but had no effect on the long‐term prognosis (beyond 3 years, Supplemental figure). Multivariate analysis showed that tumour size and AJCC stage were the risk factors for OS, while DUSP4 expression could not act as an independent prognostic factor for OS (Table S2).

**Table 1:** Correlation of DUSP4 expression with clinicopathological parameters in GC patients

Variables	Cases (*n*)	DUSP4 expression		*P*
		Low (%)	High (%)	
Age				
≤60	29	17 (58.6)	12 (41.4)	0.290
>60	60	28 (46.7)	32 (53.3)	
Gender				
Male	69	39 (56.5)	30 (43.5)	0.037
Female	20	6 (30.0)	14 (70.0)	
Tumour size (cm)				
≤3.5	17	3 (17.6)	14 (82.4)	0.020
>3.5	72	35 (48.6)	37 (51.4)	
AJCC stage				
I+II	37	17 (45.9)	20 (54.1)	0.463
III+IV	52	28 (53.8)	24 (46.2)	
Depth of invasion				
Mucosa/Submucosa	5	2 (40.0)	3 (60.0)	0.008
Muscularis/Serosa/	84	72 (85.7)	12 (14.3)	
T stage				
T1+T2	11	4 (36.4)	7 (63.6)	0.514
T3+T4	78	21 (26.9)	57 (73.1)	
N stage				
N0+N1	39	11 (28.2)	28 (71.8)	0.983
N2+N3	50	14 (28.0)	36 (72.0)	
M stage				
Negative	84	24 (28.6)	60 (71.4)	0.016
Positive	5	4 (80.0)	1 (20.0)	

### Sanguinarine inhibits proliferation and invasion of GC cells

**Figure 2:**
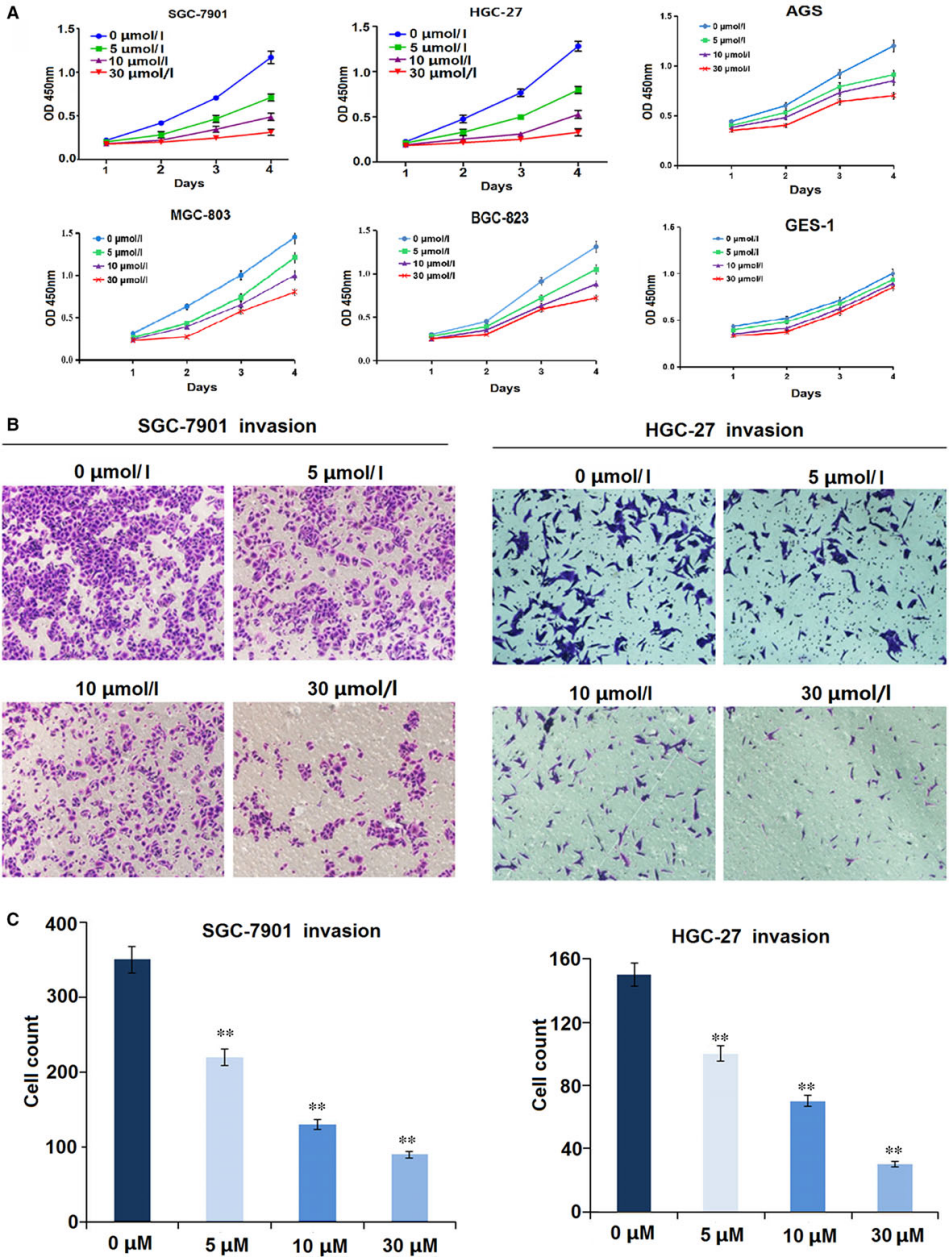
Sanguinarine inhibited GC cell proliferation and invasion. (**A**) Cell proliferative activity was evaluated by CCK‐8 assay, indicating that sanguinarine decreased cell proliferation in dose‐ and time‐dependent manners, but exerted little inhibitory effects on GES‐1 cells. (**B,C**) Cell invasive potential was decided by Transwell assay, indicating that sanguinarine reduced cell invasion in a dose‐dependent manner. **P < 0.01.

The chemical structure of sanguinarine is shown in Figure 1C. The inhibitory efficacy of sanguinarine on GC cell growth was evaluated by the CCK‐8 assay. The major characteristics of GC are its excessive local invasion and systemic metastasis. Cell invasive potential was determined by Transwell assay. As a consequence, we found that sanguinarine exerted inhibitory effects on GC cells growth, but exerted little inhibitory effects on GES‐1 cells (Fig. 2A). What is more, sanguinarine could inhibit GC cells invasion (Fig. 2B and C) in a dose‐dependent manner (**P < 0.01).

### Sanguinarine induces cycle arrest in S phase and causes apoptosis in GC cells

**Figure 3:**
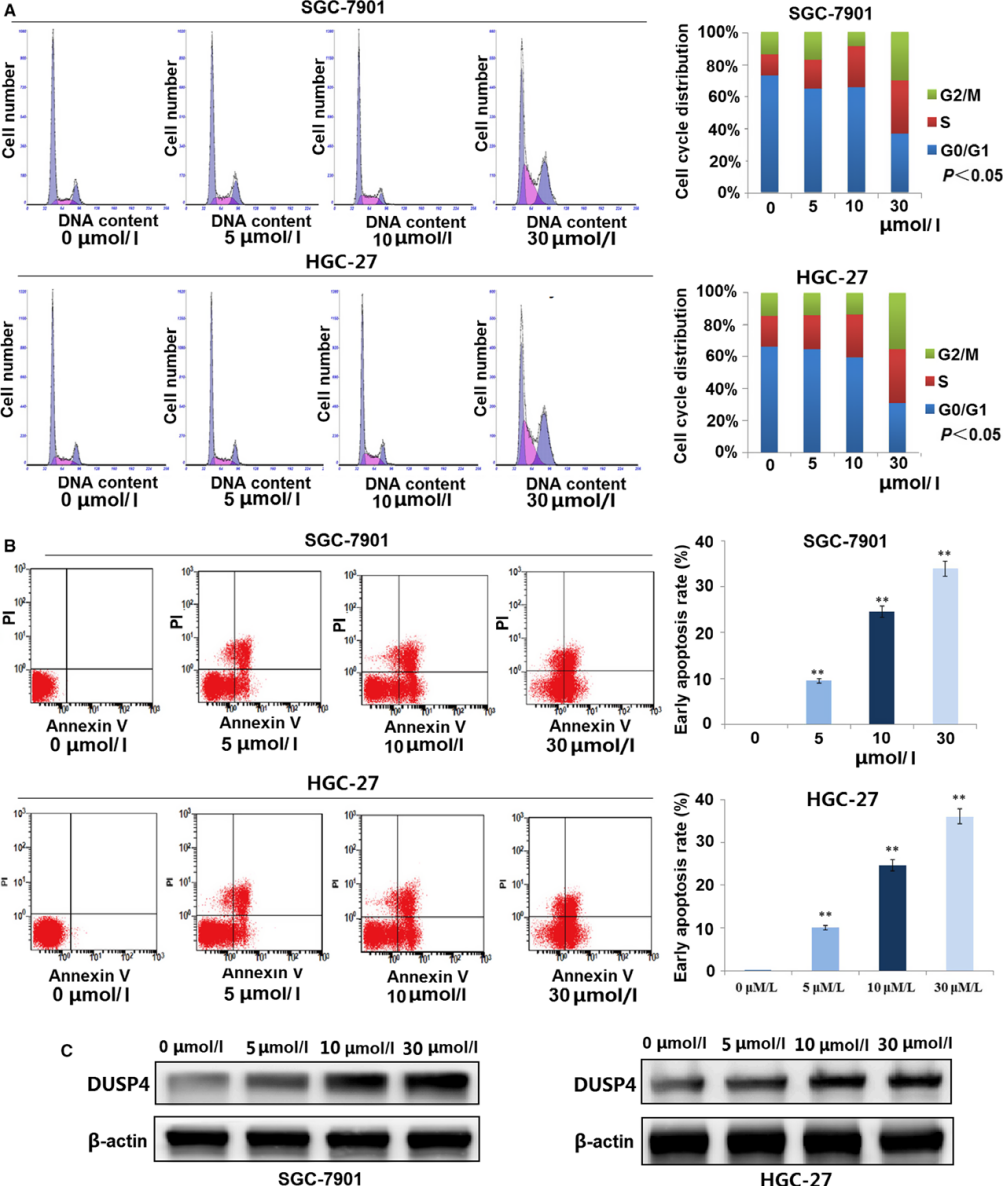
Sanguinarine induced GC cycle arrest and cell apoptosis. (**A**) Cell cycle analysis showed that sanguinarine increased GC cell proportion in cell cycle S phase in a dose‐dependent manner. *P* < 0.05. (**B**) Cell apoptotic analysis indicated that sanguinarine induced cell apoptosis in a dose‐dependent manner. **P < 0.01. (**C**) Expression of DUSP4 in GC cells treated by sanguinarine of different concentrations.

To investigate whether sanguinarine blocked cell cycle progression, SGC‐7901 and HGC‐27 cells were exposed to various concentrations of sanguinarine (0/5/10/30 μmol/l) for 24 hrs, and cell cycle analysis was conducted. We found that sanguinarine increased the percentage of GC cells in S phase in a dose‐dependent manner, but had little effects on G0/G1 or G2/M phase (Fig. 3A). The results showed that sanguinarine could inhibit DNA synthesis and thus induce cycle arrest. In addition, flow cytometry analysis showed that sanguinarine induced cell apoptosis in a dose‐dependent manner in GC cells (**P < 0.01, Fig. 3B). Western blotting showed that DUSP4 expression was raised by sanguinarine in a dose‐dependent manner, but there is little increase of protein expression in 30 μmol/l group compared with that in 10 μmol/l group (Fig. 3C).

### DUSP4 overexpression inhibits proliferation and invasion of GC cells

**Figure 4:**
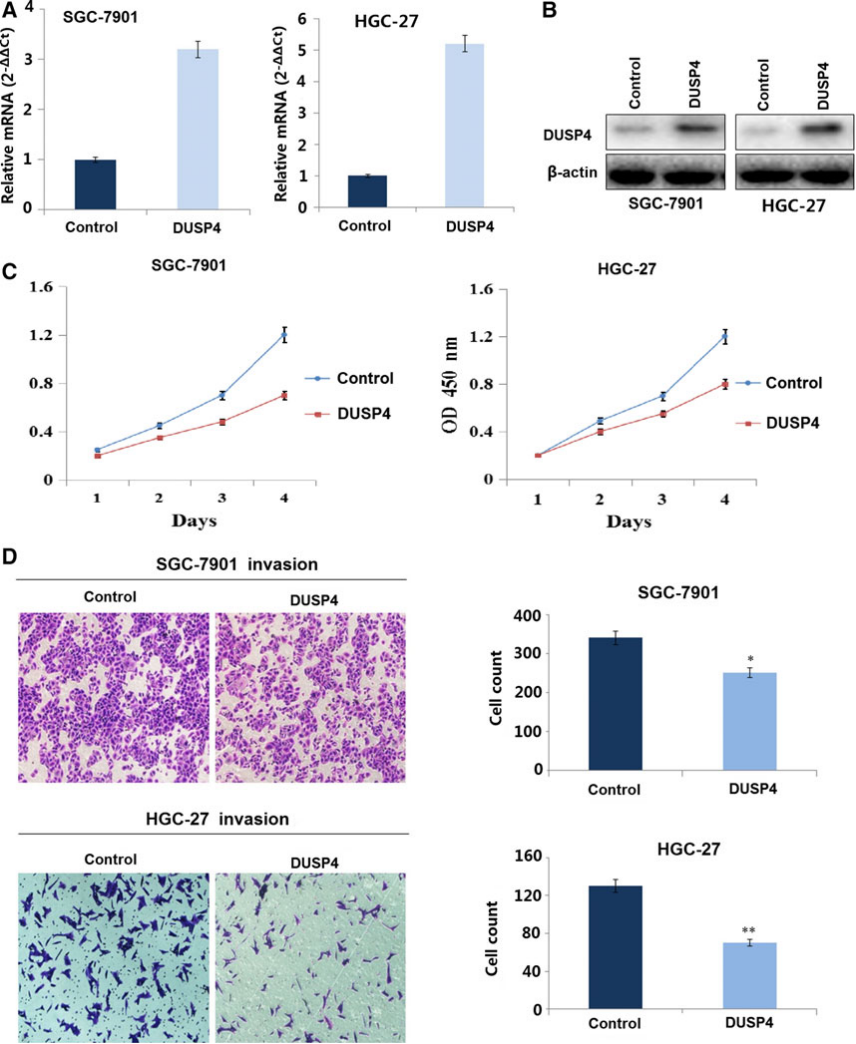
DUSP4 overexpression inhibited GC cell proliferation and invasion. (**A**) DUSP4 mRNA and (**B**) protein expression were, respectively, examined by RT‐PCR and Western blotting in SGC‐7901 and HGC‐27 cells transfected with DUSP4. (**C**) CCK‐8 assay indicated that overexpression of DUSP4 decreased cell growth after DUSP4 transfection for 24 hrs. (**D**) Transwell invasion assay showed that overexpression of DUSP4 weakened cell invasive potential compared with the NC group. *P < 0.05, **P < 0.01.

To further confirm the function of DUSP4 in GC cells, DUSP4 was stably overexpressed through a lentiviral vector in SGC‐7901 and HGC‐27 cell lines with low endogenous DUSP4 level. The DUSP4 expression level was examined by Real‐time PCR (Fig. 4A) and Western blotting analysis (Fig. 4B). To verify whether DUSP4 acted a role in GC growth and invasion, the cell proliferation and invasive potential were determined by CCK‐8 and Transwell assays. The results demonstrated that overexpression of DUSP4 significantly decreased cell proliferation (Fig. 4C) and invasive ability (Fig. 4D) compared with the control group in GC cells (**P < 0.01).

### DUSP4 knockdown attenuated sanguinarine‐induced inhibition of cell growth

**Figure 5:**
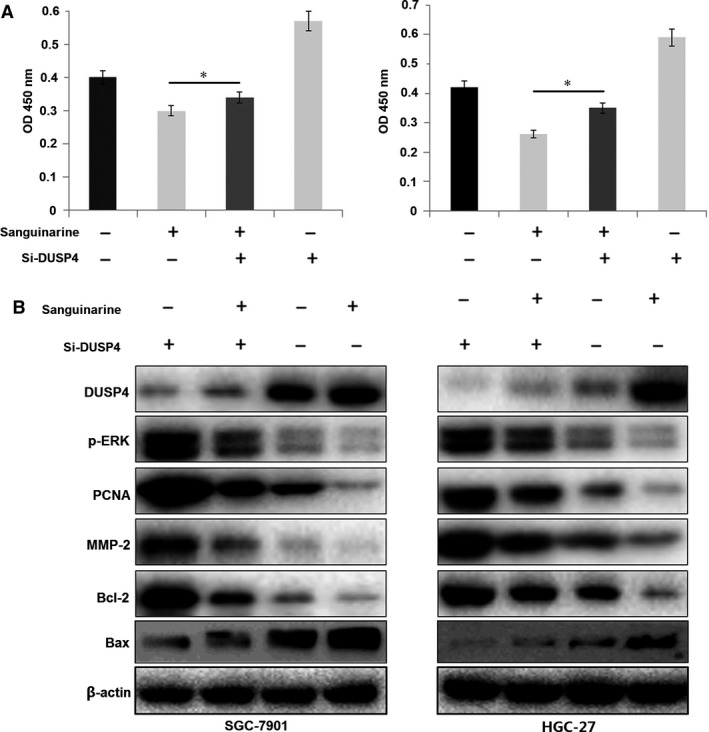
DUSP4 knockdown attenuated sanguinarine‐induced inhibition of GC growth and ERK signalling. (**A**) Lentivirus‐mediated si‐DUSP4 was used to transfect into sanguinarine (10 μmol/l)‐treated GC cells for 72 hrs, and proliferative activity of SGC‐7901 and HGC‐27 cells was evaluated by MTT assay. (**B**) The protein expression levels of DUSP4 and its downstream factors including p‐ERK, PCNA, MMP‐2, Bax and Bcl‐2 were detected by Western blotting. *P < 0.05.

**Figure 6:**
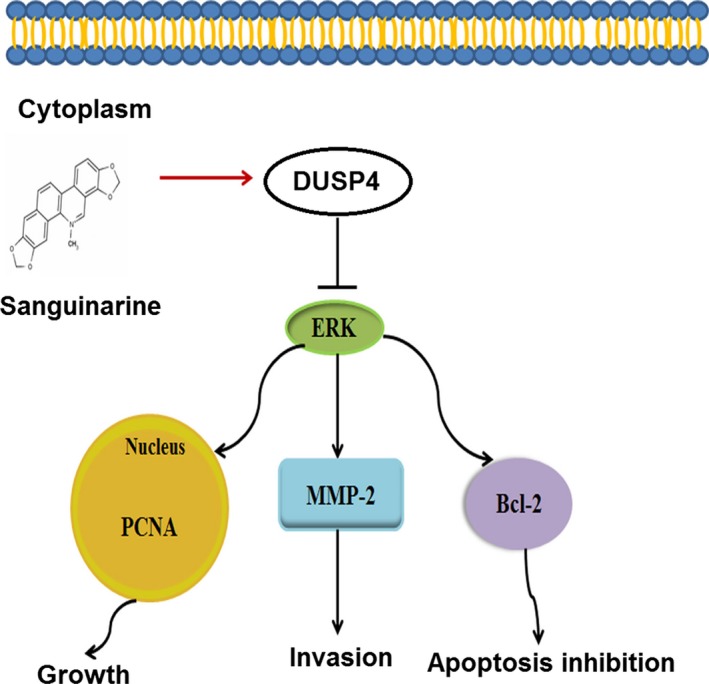
Sanguinarine inhibits growth and invasion and induces apoptosis of gastric cancer cells *via* regulation of the DUSP4/ERK pathway. The graph located in the ceiling position represents lipid bilayer membranes. The molecular formula represents sanguinarine. The blue oval represents DUSP4 protein. The green oval below represents ERK protein.

To explore the relationship between sanguinarine and DUSP4/ERK signalling, CCK‐8 proliferation assay was conducted. The results showed that DUSP4 knockdown decreased sanguinarine‐induced inhibition of GC cell proliferation (Fig. 5A). To test the molecular mechanisms of sanguinarine inhibiting GC proliferation and invasion, DUSP4 and downstream‐related proteins involved in cellular processes were detected. Western blot analysis showed that exposure to 0/5/10/30 μmol/l sanguinarine increased DUSP4 and Bax expression, but decreased the expressions of p‐ERK, and its downstream signalling molecules in GC cells, such as PCNA, MMP‐2 and Bcl‐2, involved in cell proliferation, invasion and anti‐apoptosis, respectively (Fig. 5B). DUSP4 knockdown decreased the effects of sanguinarine on the expression levels of DUSP4 and downstream factors, suggesting that sanguinarine down‐regulated the expressions of activated ERK and its downstream signalling molecules through increasing DUSP4 expression in a dose‐dependent manner, and therefore exerted the antitumour effects (Fig. 6).

## Discussion

Although chemotherapy occupies the main position in the treatment of human cancer, the response rates of most chemotherapeutic agents are low. In addition, drug resistance and adverse reactions often occur, reducing the survival quality of patients with cancer and hindering the effective application of these agents. Natural products and their derivatives have caught the attention of chemists and pharmacologists, on account of their promising therapeutic applications and rich structural diversity 18, 26. Medical scientists devote themselves to developing new, safe and effective drugs from natural products for cancer therapy 27, 28. Sanguinarine is a bioactive benzophenanthridine alkaloid, which is used for its anti‐inflammatory, antimicrobial, antioxidative and anaesthesia effects on the central nervous system 21. Sanguinarine has been proven to possess anticancer activity, although the underlying mechanisms are not elucidated. Herein, we reported that sanguinarine, a natural product isolated from the Papaveraceae family, exhibited potent anticancer activity. It exerted anticancer effects on SGC‐7901 and HGC‐27 GC cells by inhibiting cell proliferation and invasion, and inducing cell apoptosis in a dose‐dependent manner and provided the therapeutic strategy for the GC treatment.

Currently, the roles of DUSP4 in cancers remain controversial. Some studies showed DUSP4 could promote the progress of colorectal cancer 29, 30, 31 and breast malignancy 32, while some researches showed DUSP4 could inhibit the tumour development in colorectal cancer 33, breast cancer 17, 34, diffuse large B‐cell lymphoma 35, laryngeal cancer 36, head and neck squamous cell carcinoma 37. However, few studies have been reported about DUSP4 expression and GC. DUSP4 knockdown enhances ERK activation 17, 38, while DUSP4 overexpression suppresses ERK activation 39. In our experiments, sanguinarine increased DUSP4 expression, thus causing the reduction in p‐ERK‐regulating genes, such as PCNA, MMP‐2, and Bcl‐2, respectively, involved in cell proliferation, invasion and anti‐apoptosis 40, 41, 42, 43, 44. There were many genes affecting the progress of GC, and our multivariate analysis showed that DUSP4 alone might be not enough to change the OS of patients with GC. Therefore, there was no significant correlation between DUSP4 expression and OS, but decreased DUSP4 expression was correlated with gender, tumour size, depth of invasion and distant metastasis, suggesting that DUSP4 might be implicated in the development of GC.

The ability of ERK to regulate cell proliferation and apoptosis provides a promising target for cancer therapy 45, 46. Aberrant activation of the Raf/MEK/ERK, which drives uncontrolled tumour cell proliferation and survival, is detected in a variety of human cancers 47, 48, 49, 50. Cell surface receptor tyrosine kinases, or oncogenic alterations of RAF, or its upstream activators RAS, contribute to aberrant activation of this pathway 51, 52, 53, 54. DUSP4, a dual‐specificity phosphatase, can inactivate Raf/MEK/ERK signalling pathway through dephosphorylating p‐ERK. Moreover, we examined the expression level of DUSP4 in GC tissues and found that DUSP4 was negatively correlated with the distant metastasis in patients with GC. Overexpression of DUSP4 inhibited GC cell proliferation and invasion, while its knockdown weakened the antitumour activity of sanguinarine. Therefore, DUPS4 might function as a tumour suppressor and mediate the inhibitory effects of sanguinarine on GC cells.

In summary, our study has shown that sanguinarine has potent antitumour activity *in vitro* by inhibiting cell proliferation and invasion, and inducing cell apoptosis and cycle arrest through activation of the DUSP4 pathway. But, there are several deficiencies in these studies. Our studies mainly focus on the regulation of sanguinarine on the DUSP4/ERK signalling in GC cells, and further verification in animal models is needed.

## Conflict of interests

No conflict of interests to disclose.

## Supporting information


**Table S1** Primer sequences for detection of mRNA expression.
**Table S2** Effect of variables on overall survival in gastric cancer.
**Table S3** DUSP4 expression in GC and ANCT.
