# Supplementary material for: Sanguinarine inhibits growth and invasion of gastric cancer cells via regulation of the DUSP4/ERK pathway
Source: J Cell Mol Med. 2016 Dec 13;21(6):1117–27. doi: 10.1111/jcmm.13043 (PMC5431127; doi:10.1111/jcmm.13043)
Supplement: Supplementary file 1 — Table S1 Primer sequences for detection of mRNA expression. Table S2 Effect of variables on overall survival in gastric cancer. Table S3 DUSP4 expression in GC and ANCT. [file JCMM-21-1117-s001.docx]

Supplementary Table 1 Primer sequences for detection of mRNA expression

| Name | Forward primer (5′→3′) | Reverse primer (5′→3′) |
| --- | --- | --- |
| DUSP4 | GTACAAGTGCATCCCAGTGGA | CTTCATCATCAGGTAGGCCAGG |
| GAPDH | TCGTGCGTGACATTAAGGAG | ATGCCAGGGTACATGGTGGT |

Supplementary Table 2 Effect of variables on overall survival in gastric cancer

| Variables | Univariable analysis |  | Multivariable analysis |
| --- | --- | --- | --- |
|  | HR (95% CI) | *P* value | HR (95% CI) |
| Tumor size (≤32 vs. >32 cm^3^) | 2.274 (1.364-3.790) | P<0.001 | 2.516 (1.485-4.262) |
| AJCC stage ( I, II versus III, IV) | 2.686 (1.545-4.671) | *P*<0.001 | 2.693 (1.536-4.723) |
| DUSP4 expression (low vs. high) | 0.725 (0.438-1.200) | 0.205 | 0.713 (0.429-1.185) |

Supplementary Table 3 DUSP4 expression in GC and ANCT

|  | DUSP4 expression |  |
| --- | --- | --- |
|  | Low (%) High (%) | *P* |
| GC  ANCT | 44 (49.4%) 45 (50.6%)  24 (27.0%) 65 (73.0%) | 0.001 |
